# Resistance training in overweight women on a ketogenic diet conserved lean body mass while reducing body fat

**DOI:** 10.1186/1743-7075-7-17

**Published:** 2010-03-02

**Authors:** Pal T Jabekk, Ingvild A Moe, Helge D Meen, Sissel E Tomten, Arne T Høstmark

**Affiliations:** 1 Department of Sport Medicine, Norwegian School of Sport Sciences, Sognsveien 220, Oslo, Norway; 2 Section of Preventive Medicine and Epidemiology, University of Oslo, Norway; 3 Department of Physical Performance, Norwegian School of Sport Sciences, Sognsveien 220, Oslo, Norway

## Abstract

**Background:**

The aim of the present study was to compare the effects of 10 weeks resistance training in combination with either a regular diet (Ex) or a low carbohydrate, ketogenic diet (Lc+Ex) in overweight women on body weight and body composition.

**Methods:**

18 untrained women between 20 and 40 years with BMI ≥ 25 kg*m^-2 ^were randomly assigned into the Ex or Lc+Ex group. Both groups performed 60-100 min of varied resistance exercise twice weekly. Dietary estimates were based on two 4-day weighed records. Body composition was estimated using Dual Energy X-ray Absorptiometry. Fasting blood samples were analyzed for total-, HDL- and LDL-cholesterol, triacylglycerols, and glucose.

**Results:**

16 subjects were included in the analyses. Percentage of energy (En%) from carbohydrates, fat and protein was 6, 66, and 22 respectively in the (Lc+Ex) group and 41, 34, 17 in the Ex group. Mean weight change (pre-post) was -5.6 ± 2.6 kg in Lc+Ex; (p < 0.001) and 0.8 ± 1.5 kg in Ex; (p = 0.175). The Lc+Ex group lost 5.6 ± 2.9 kg of fat mass (p = 0.001) with no significant change in lean body mass (LBM), while the Ex group gained 1.6 ± 1.8 kg of LBM (p = 0.045) with no significant change in fat mass (p = 0.059). Fasting blood lipids and blood glucose were not significantly affected by the interventions.

**Conclusion:**

Resistance exercise in combination with a ketogenic diet may reduce body fat without significantly changing LBM, while resistance exercise on a regular diet may increase LBM in without significantly affecting fat mass. Fasting blood lipids do not seem to be negatively influenced by the combination of resistance exercise and a low carbohydrate diet.

## Background

The prevalence of obesity is increasing and there is a need for safe and effective long term weight loss strategies. Low carbohydrate ketogenic diets have the potential to be one such strategy and recent research supports the notion that these diets may be as effective or more effective than regular calorie restricted diets, both in reducing body weight and in improving metabolic disorders [1-4]. Overweight and obesity are commonly associated with a cluster of metabolic risk factors related to insulin resistance [5]. Carbohydrate restricted diets seem to improve glycemic control and other features of the metabolic syndrome, and are generally found to beneficially influence risk factors for cardiovascular diseases [5,6].

A 2006 Cochrane meta - analysis [7] concluded that a low carbohydrate diet without an imposed energy restriction, appears to be at least as effective as low fat energy restricted diets in inducing weight loss during time periods up to 1 year. A number of other studies has confirmed this finding [8-11]. A more recent systematic review [4] concluded that low carbohydrate/high protein diets are more effective during a 6 month period and as effective as low-fat diets in reducing weight and cardiovascular disease risk up to 1 year [4]. In contrast, Sacks et al [12] recently observed no difference in weight loss after introducing four diets differing in carbohydrate as well as fat and protein content in a 2 year study. However, the dietary goals were only partly achieved and the lowest average intake of carbohydrate was found to exceed 42 En% both at 6 and 24 months

A greater weight loss with a carbohydrate restricted diet compared to a fat restricted diet has been shown to persist under isocaloric conditions [13]. This finding has been classified as a decreased caloric efficiency or a metabolic advantage [13-15], and may be an additional positive feature of carbohydrate restriction. The mechanisms for a metabolic advantage is thought to be related to the inefficiency of various metabolic cycles and especially protein turnover [13].

An important issue related to the effectiveness of any weight reducing regime is the impact on body composition, thus the effectiveness of different diets may be difficult to compare based on body weight reduction alone. Loss of lean body mass seems to be a common observation in most types of weight loss strategies [16-19]. In one study [20], 8 weeks of regular calorie restricted diet with 54 - 60% of energy from carbohydrate, caused a significant decrease in body weight in both men and women. However, the loss of fat mass in the female participants was accompanied by a large loss of LBM and did not cause any significant decrease in the percentage of body fat. There are conflicting results concerning the impact of low carbohydrate diets vs. low fat diets on body composition during weight reduction although several results indicate an advantage of low carbohydrate diets in maintaining or increasing LBM during dieting [21-23]. According to a meta - regression by Krieger et al [14], low carbohydrate diets are associated with a greater total loss of fat free mass, but also greater loss of fat mass and total body weight and thus a greater lowering of body fat percentage, compared with diets higher in carbohydrate.

The most effective tool for increasing or maintaining lean body mass is resistance exercise training [24]. Resistance exercise has been shown to limit the loss of LBM [25-27] during weight loss, although aerobic exercise has been shown to have similar effects [19]. Maintaining or increasing LBM is of importance for maintaining an adequately high metabolism and reduce the tendency to regain weight [25] and for maintaining adequate body function with aging [28,29]. In addition, resistance exercise also has the potential to improve metabolic disorders associated with overweight and poor glycemic control [30-32], to reduce the requirement for diabetes medications and to reduce abdominal adiposity and systolic blood pressure [30]. Resistance exercise has also been associated with improvements in cardiovascular disease (CVD) risk factors in the absence of significant weight loss [25].

A comparatively large loss of lean body mass to fat mass has been proposed as a possible explanation for the frequently observed long term failure of various weight loss regimes [33]. As loss of fat may seem to be greater when intake of carbohydrates is restricted compared to fat restriction [9,34-38], and as resistance exercise seems to be the most effective tool for increasing muscle mass, a combination of the two strategies may have the potential for weight loss with a favorable change in body composition.

However, with limited glycogen stores resulting from a carbohydrate restricted diet, physical performance and especially anaerobic performance may be attenuated [39], and little is known of the ability to perform severe fatiguing exercise on a low carbohydrate ketogenic diet. Even less is known of the ability to perform enduring anaerobic exercises like heavy resistance exercise.

The aim of the present study was therefore to investigate how resistance exercise in combination with a low carbohydrate ketogenic diet would influence body weight and composition compared to the same resistance exercise in combination with a regular diet, and if the two regimes might have any effects on fasting blood lipids.

## Methods

### Subjects

Overweight female volunteers were recruited from the Oslo area, through newspaper, webpage and local community notices. Participants were between 20 - 40 years, with a BMI ≥ 25 kg/m^2^. The participants had not previously been using low carbohydrate diets, and had not participated in regular resistance exercise during the last 6 months. Exclusion criteria included pregnancy or planned pregnancy, cardiovascular disease, diabetes, kidney diseases, physical disabilities that would hinder resistance exercise or medications that could potentially influence exercise or blood variables. Eighteen subjects fulfilled the criteria and were randomized to either resistance exercise combined with a regular diet (Ex) or resistance exercise in combination with a low carbohydrate ketogenic diet (Lc+Ex).

Prior to the study a written informed consent was obtained from all participants. In addition, all subjects provided a written consent from their personal physician. The study was approved by The Regional Ethics Committee of Norway (ref.nr: S-08251a).

### Final study group

One participant in the Lc+Ex group did not complete the study due to personal reasons. Another participant in the Ex group had reduced her carbohydrate intake to a level that induced ketosis. The dietary records from this participant further supported this finding and revealed a dietary pattern similar to that of the Lc+Ex group. As a consequence, data from this participant were excluded. Thus, the final analyses included 16 participants.

### Exercise intervention

All exercise was performed at the Norwegian School of Sport Sciences (NSSS) and every exercise session was supervised by at least one qualified instructor. During a pre-session all subjects were instructed in the exercise program. During this session the individual 12 repetition maximum (12 RM) in all exercises was assessed. Eight exercises were performed after an initial low-intensity warm up (10 min) on a cardio machine. The exercises consisted of supine leg press, seated leg extension, seated leg curl, seated chest press, seated rowing, seated shoulder press, seated pull down and standing biceps curl. As far as possible the exercises were conducted in the given order. During the first five weeks of the intervention, all exercises were performed at 12 RM with three sets on leg exercises and one set on upper body exercises. After the initial five weeks, resistance loads were increased to 8 RM and an additional set was added to the upper body exercises. Each exercise included two warm - up sets with a resistance equivalent to approximately 25% and 50% of the maximum load (i.e. 12 RM & 8 RM). Resting periods of approximately 90 s between all sets were encouraged. All participants were advised not to participate in other forms of resistance exercise during the study period, and to otherwise maintain their usual level of physical activity throughout the study.

### Diet intervention

Participants in both groups were given a daily multivitamin mineral supplement. Each group was counseled in exercise and diet during a single group session. Participants in the Lc+Ex group were given information on the rationale and implementation of the dietary intervention and were given a commercially available book on a low carbohydrate ketogenic diet (Slank med ketolysekuren: en enklere vei til et lettere liv; "Slim with ketolysis: A simpler way to a lighter life") [40] in addition to handouts. The preparation of food was self administered. No restrictions were made regarding energy content, fat and protein content or fatty acid composition. The only restriction was on intake of carbohydrates, and the goal was to restrict carbohydrate intake until ketone bodies were detectable in the urine. The presence of ketone bodies was detected with a semi quantitative method using urine reagent strips (Ketolyse AS). The reagent strips indicated the concentration of acetone and acetoacetic acid in the urine. Participants were told to start the intervention with less than 20 g carbohydrate per day and to gradually increase the ingestion of carbohydrates at their convenience, as long as they maintained a color change on the urine reagent strips. The diet intervention was thus defined as a carbohydrate restriction that caused positive tests for urinary ketone bodies. Participants were told that they could consume unlimited amounts of meats, fish, eggs, cheeses, margarines, butters and oils. They were further instructed to add low carbohydrate food to their diet as they saw fit.

### Body mass and body composition

Body weight and composition was determined by Dual Energy X - ray Absorptiometry (DEXA) total body scanner (Prodigy Lunar, Scanex Medical Systems A/S). The dexa scanner partitions body mass into three components: fat, lean soft tissue, and bone mineral using two x-rays. Height was measured to the nearest 0.5 cm using a wall mounted stadiometer.

### Blood sampling

Venous blood samples were drawn in the morning after an overnight fast, at baseline, halfway into the intervention and the week following the intervention period. Carbohydrate restriction was maintained until the last blood sample was collected. Samples were collected at the Norwegian School of Sport Sciences by qualified health personnel, centrifuged and sent to a certified medical laboratory (Fürst Medical Laboratory, Oslo, Norway). Serum blood samples were analyzed for total-, HDL- and LDL-cholesterol, triacylglycerols (TAG), and glucose using automated photometric techniques (Modular P Roche; Roche Diagnostics). The baseline sample was also analyzed for creatinine.

### Diet analyses

The subjects weighed and recorded all their intake of food and drinks on four consecutive days (Wednesday to Saturday) on two occasions, using an electronic weight scale (precision 2 g). When special recipes were supplied by the subjects, each ingredient was entered separately. The dietary data were analyzed using the Norwegian Nutritional Computer Analysis Program (Mat på Data v.5) which is based on the Norwegian Food Composition Tables [41]. Mean daily intakes were estimated to be 1/4 of the individual total recorded amounts. The diet recordings were performed during the weeks 4 and 7 of the study. The mean of the two recordings were used as an estimate of dietary intake during the intervention period.

### Statistical analyses

Statistical analyses were performed using SPSS for WINDOWS software (version 14.0; SPSS Inc.). Changes from baseline were assessed using paired sample t - test. Differences in group means were assessed using independent sample t - test. Correlation analysis was used to determine relations between variables. Statistical significance was set at p ≤ 0.05. Unless otherwise indicated, values are presented as mean values ± SD.

## Results

Attendance at the exercise sessions averaged 18 out of 20 sessions and did not differ between groups.

### Changes in body weight and body composition (Table 1)

**Table 1:** Anthropometric measures

	(Lc+Ex) (n = 8)			Ex (n = 8)		
		
	Baseline	Week 10	Change	Baseline	Week 10	Change
**Weight (kg)**	95.6 ± 9.1*	90.0 ± 8.8^†^	-5.6 ± 2.6**	86.1 ± 8.4	87.0 ± 7.9	0.8 ± 1.5
**LBM (kg)**	48.5 ± 3.9*	48.5 ± 3.9	0.1 ± 1.7	43.3 ± 3.5	44.8 ± 3.7^†^	1.6 ± 1.8
**Fat mass (kg)**	44.0 ± 8.6	38.4 ± 9.1^†^	-5.6 ± 2.9*	40.2 ± 6.3	39.5 ± 6.6	-0.6 ± 0.8
**Fat %**	47.3 ± 5.8	43.7 ± 7.0^†^	-3.6 ± 2.5*	47.9 ± 3.4	46.7 ± 4.2^†^	-1.3 ± 1.3
**BMI**	32.9 ± 4.5	31.0 ± 4.5^†^	-1.9 ± 0.8**	31.7 ± 4.2	32.0 ± 4.0	0.3 ± 0.5

Mean values ± SD. *p ≤ 0.05 vs Ex. **p ≤ 0.001 vs Ex. ^†^Significant change from baseline p ≤ 0.05.

At baseline there were significant differences between groups in body weight (p = 0.04) and LBM (p = 0.014). These differences were not significant when corrected for differences in height i.e. BMI (Table 1).

**Figure 1:**
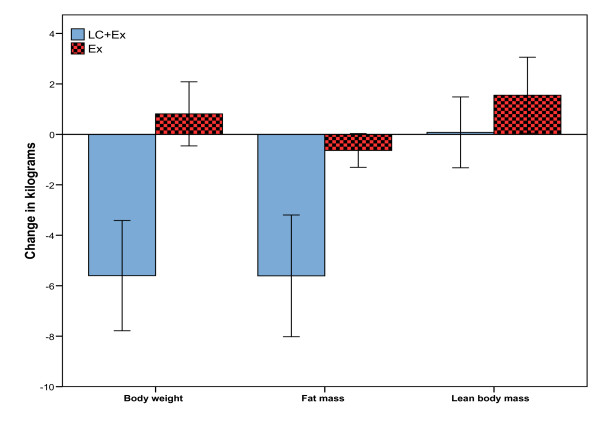
**Effect of resistance exercise in combination with a regular diet (Ex) or resistance exercise in combination with a ketogenic diet (Lc+Ex)**. Group changes from baseline in body weight and composition. Error bars indicate 95% confidence interval.

All subjects in the Lc+Ex group lost weight. While the Lc+Ex group in average lost 5.6 ± 2.6 kg body mass (p < 0.001), the Ex group had a non significant increase in body weight 0.8 ± 1.5 kg (p = 0.175) (figure 1). Body weight change was significantly different between groups (p < 0.001). The Ex group lost on average 0.6 kg of fat mass (p = 0.059) from 40.2 ± 6.3 kg to 39.5 ± 6.6 kg and had a significant increase in LBM (1.6 ± 1.8 kg, p = 0.045). In the Lc+Ex group, mean fat mass was reduced by 5.6 ± 2.9 kg (p < 0.001) from 44.0 ± 8.6 kg to 38.4 ± 9.1 kg. However, individual fat loss varied greatly (Range: - 2.2 kg to - 10.5 kg).

**Figure 2:**
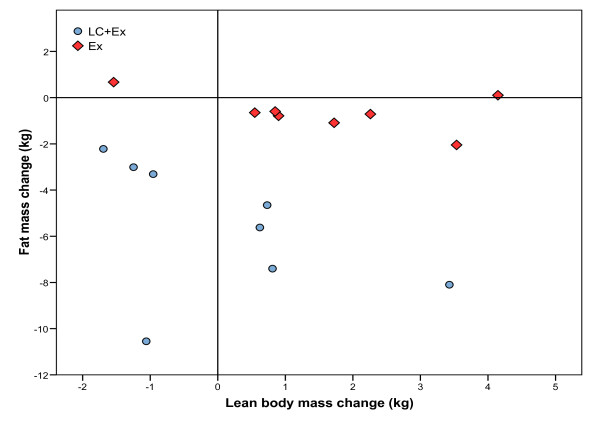
**Effect of resistance exercise in combination with a regular diet (Ex) or resistance exercise in combination with a ketogenic diet (Lc+Ex)**. Individual changes in body fat mass and lean body mass.

Mean LBM change in the Lc+Ex group was not significant (0.1 ± 1.7 kg, p = 0.897) and although a significant increase was found within the Ex group, there was no significant difference in LBM change between groups (p = 0.113). The large individual differences in body fat and LBM change are presented in figure 2. Both intervention groups experienced a favorable change in body composition as percent body fat decreased from 47.3 ± 5.8 to 43.7 ± 7.0 kg (p = 0.006), in the Lc+Ex group and from 47.9 ± 3.3 to 46.7 ± 4.1 kg (p = 0.028), in the Ex group.

### Dietary nutrient intakes (Table 2)

**Table 2:** Dietary intake from two 4 d food records

NUTRIENT	(Lc+Ex) (n = 8)	Ex (n = 8)
**Energy (kJ)**	7349 ± 1438	8260 ± 1684
**Fat (g)**	131 ± 27*	76 ± 18
**Fat (% of energy)**	66 ± 5*	34 ± 3
**Carbohydrate (g)**	23 ± 10*	196 ± 35
**Carbohydrate (% of energy)**	6 ± 3*	41 ± 4
**Protein (g)**	95 ± 26	79 ± 11
**Protein (% of energy)**	22 ± 4*	17 ± 2
**Dietary fiber (g)**	12 ± 6*	20 ± 5
**Alcohol (% of energy)**	5 ± 5	7 ± 6

Mean values ± SD. *p ≤ 0.05 vs Ex.

The dietary records revealed no significant group difference in the total energy intake (p = 0.264). Fat intake was significantly higher (p ≤ 0.001), and carbohydrate was significantly lower in Lc+Ex (p ≤ 0.001). Mean carbohydrate intake in the Lc+Ex group was 23 g/day, i.e. 5.6% of the energy intake. This level was sufficiently low to induce ketosis as indicated by the urine reagent strips. Total protein intake (g/day) did not differ between groups (p = 0.133), but did differ significantly expressed as a percentage of total energy (p = 0.007).

In the Lc+Ex group there was a significant negative correlation between the reduction in fat mass and dietary fat intake in grams (r = -0.714, p = 0.047) and also between the reduction in fat mass and energy intake (r = -0.810, p = 0.015). In the Ex group there was a significant correlation between carbohydrate intake in grams and LBM increase (r = 0.707, p = 0.05). Pooled data from the two groups did not show significant correlations between energy intake and reduction in body fat mass (r=-0.315).

### Fasting blood lipids and glucose (Table 3)

**Table 3:** Fasting blood glucose and serum lipids

	(Lc+Ex) (n = 8)					Ex (n = 8)			
			
	Baseline	Week 5	Week 10	Change pre-post		Baseline	Week 5	Week 10	Change pre-post
**Glucose (mmol/l)**	4.9 ± 0.3	5.4 ± 0.9	5.0 ± 0.3	0.1 ± 0.3		5.0 ± 0.3	4.9 ± 0.5	5.1 ± 0.5	0.1 ± 0.4
**Total-C (mmol/l)**	5.6 ± 1.1	5.8 ± 1.4	5.7 ± 1.2	0.1 ± 1.0		5.0 ± 0.7	5.0 ± 0.8	4.8 ± 0.7	-0.2 ± 0.6
**LDL-C (mmol/l)**	3.3 ± 0.9	3.6 ± 1.2	3.5 ± 1.1	0.2 ± 0.9		2.9 ± 0.5	2.9 ± 0.6	2.7 ± 0.6	-0.1 ± 0.4
**HDL-C (mmol/l)**	1.6 ± 0.5	1.5 ± 0.4	1.5 ± 0.4	-0.1 ± 0.2		1.4 ± 0.2	1.4 ± 0.2	1.4 ± 0.2	0.1 ± 0.2
**TAG (mmol/l)**	1.2 ± 0.8	1.0 ± 0.3*	0.9 ± 0.3*	-0.3 ± 0.6		1.4 ± 0.5	1.4 ± 0.4	1.3 ± 0.4	-0.1 ± 0.3

Mean values ± SD. *p ≤ 0.05 vs Ex.

Fasting values of blood glucose, total-, HDL- and LDL-cholesterol, and triacylglycerols (TAG) did not differ significantly between the groups at baseline. At midpoint and end point, the Lc+Ex had significantly lower fasting TAG levels (p = 0.033 and p = 0.044 respectively). Neither the combination of low carbohydrate intake and exercise nor exercise with a regular diet had any significant effects on the measured blood variables.

### Adverse effects

Adverse effects related to the two regimes were not observed. One participant in the Lc+Ex group experienced low back pain due to a previous injury when performing the leg press exercise. This participant refrained from this exercise for most of the intervention. Some of the participants experienced stomach discomfort when taking the multi - vitamin mineral supplements on an empty stomach. This was corrected after the participants were encouraged to take the supplements with a meal.

## Discussion

### Body composition

The observation that resistance exercise in combination with a low carbohydrate diet resulted in reduced body weight and body fat as compared with similar exercise while on a regular diet is in accordance with previous studies showing a large loss of weight and body fat when carbohydrate intake is reduced and maintained at a low level [34-37,42-48].

This study do not clarify whether the observed results can be attributed to energy restriction in general or to carbohydrate restriction *per se*. Whatever the mechanisms might be, the subjects in the Lc+Ex group reduced body fat while maintaining lean body mass. Similar results have also been obtained using resistance exercise in combination with a regular energy restricted diet [27]. However, a decreased feeling of hunger seems to be an advantage with the low carbohydrate diet [49-51], an effect attributed to increased levels of ketone bodies [50], reduced levels of neuropeptide Y and leptin levels and decreased insulin levels [1]. Carbohydrate restriction will reduce glucose availability and lower insulin output, and may increase the concentration of counter regulatory hormones such as catecholamines and glucagon, thereby promoting adipose tissue lipolysis [52] and a shift in metabolism from fat storage to fatty acid release and oxidation [1]. In support of this, Volek et al [22] reported that serum insulin levels were inversely related to loss of body fat in a 6 week carbohydrate restricted diet trial, and approximately 70% of the variability in fat loss was accounted for by lower serum insulin levels.

Theoretically, carbohydrate driven de novo synthesis of fatty acids in the liver should be reduced by carbohydrate restriction, thereby resulting in reduced hepatic formation of TAG and very low density lipoproteins, with ensuing reduced uptake and esterification in adipose tissue [53]. Also, decreased glucose driven formation of glycerol-3-phosphate in the fat cell should reduce adipose tissue TAG production and storage [15]. Our findings seem to support this line of reasoning since there was a significant loss of adipose tissue mass in subjects ingesting the low carbohydrate diet.

In the Lc+ Ex group no increase in mean LBM could be detected in spite of the 10 weeks of resistance training. On the other hand, there was no loss of LBM in our study, unlike results from earlier weight loss studies [14]. Although low carbohydrate diets seem to reduce the amount of LBM lost with weight reduction [54], some reduction of LBM is still expected [9,34-37,47,55]. Maintenance of, or increase in LBM with resistance exercise has previously been demonstrated in females on calorie restricted diets [26,27,56,57]. The maintenance of LBM in the present trial may therefore presumably be attributed to the resistance training.

The mechanisms behind a lower increase in LBM when on a low carbohydrate diet compared to a diet higher in carbohydrate may in part be explained by a lower energy intake and lower insulin levels. In the Ex group in the present study, there was a significant positive correlation between carbohydrate intake and the increase in LBM. Unfortunately, we do not have data on insulin. Conceivably, a higher carbohydrate intake should give higher insulin levels and thereby stimulate anabolic processes like formation of glycogen, triacylglycerols and proteins [58] and reduce catabolic ones, e.g. lipolysis in fat tissue.

A second explanation for the lack of increased LBM in the Lc+Ex group may be the use of skeletal muscle protein for fuel. Dietary induced ketosis increases the use of amino acids for glucose production [59]. Although no correlation between protein intake and LBM change was found in our study, an association between low protein intake and lean body mass loss has been found in both low carbohydrate diets [14] as well as low calorie diets [18].

Fleck and Kraemer have summarized the findings of 29 studies measuring changes in lean body mass from resistance exercise in untrained individuals [60]. Average increase in LBM constituted 2 kg in 14 weeks, or 0.06 kg of LBM per exercise session. In the studies using only female participants the equivalent number was 0.04 kg LBM increase per session. Our Ex group experienced an average LBM increase of 1.55 kg in 10 weeks or 0.08 kg per exercise session, which is in accordance with, although somewhat higher, than previously reported.

Exercise is in itself a valuable addition to weight loss diets. Meckling and Sherfey showed an added effect of exercise in inducing weight loss with both a low carbohydrate and a high carbohydrate diet. Best results on weight loss were achieved using a combination of exercise and a high protein, low carbohydrate diet [61], thus confirming the findings of Layman et al [62] who also showed that a diet with higher protein and reduced carbohydrate content combined with exercise additively improved body composition during weight loss. On the basis of these studies it seems that carbohydrate restriction in combination with exercise and resistance exercise in particular, offers an advantage over both low fat diets with exercise and low carbohydrate diets without exercise, in improving body composition.

Although there were large individual differences in body composition changes in our study, several subjects experienced very large changes in body composition in a short time period. If the details surrounding the individual differences in response to a combination of carbohydrate restriction and resistance exercise can be more clearly understood, this strategy may prove to be of great importance in the management of overweight and obesity. Also, a change in body composition may be more favorable than weight loss. Resistance exercise is in itself effective for improving body composition and is thus of importance for the treatment of obesity and its related metabolic disorders. It is, however, difficult to quantify the need for muscle mass retention. A small decrease in total muscle mass may not necessarily be considered negative. Obese individuals often have a larger total LBM than leaner counterparts. Loss of body weight will also reduce the load on the musculoskeletal system. With regard to muscle functionality and metabolic role, the quality of muscles may be considered more important than size.

### Performance

The attendance at the exercise sessions did not differ between the two groups. In addition the instructors did not observe any group differences in exercise intensity or the ability to perform at the requested loads. We therefore find it less likely that differences in exercise intensity would account for the between group differences in LBM change.

Low carbohydrate diets may result in decreased anaerobic performance [39,63], but several studies have observed no decrements in aerobic exercise with carbohydrate restricted diets [64]. Indeed, sustained aerobic performance might be beneficially influenced by a high fat diet [65-67]. White et al found a positive correlation between rating of perceived exertion and blood ketones [68]. In contrast, Brinkworth et al [37] recently observed no correlation between blood ketones and ratings of perceived exertion in their 8 week study, and observed no detrimental effects on maximal or submaximal markers of aerobic exercise or muscle strength, compared to an isocaloric high carbohydrate diet.

Unfortunately, performance ability was not measured objectively in the present study, and the possibility of reduced performance with carbohydrate restriction cannot be excluded.

### Blood lipids and glucose

As there were no significant effects on fasting serum lipids in subjects performing resistance exercise while on a ketogenic diet, and since carbohydrate restriction generally seem to improve the fasting lipid profiles [5], we would not expect harmful long term effects of the current regime on the fasting concentration of the measured blood lipids. It should be kept in mind, however, that the 24 hour serum lipid load may increase in subjects ingesting a high fat diet in spite of lower fasting levels, since the fasting concentration of TAG is the lowest value during the day on a high fat diet, while it is the highest one on high carbohydrate diet [69]. On the other hand, carbohydrate restriction may lower both fasting and non-fasting serum triglycerides [70] and studies of postprandial lipemia have shown a strong correlation between postprandial triglyceride levels and the level of fasting triglycerides [71]. Additionally, it is well known that the concentration of plasma free fatty acids (FFA) increases on a ketogenic diet [72], and high FFA levels may be an important cardiovascular risk factor [73], especially if sustained. However, plasma FFA levels were not determined in the present trial. In any instance, we consider the exercise part of the present trial to have positive health effects. Both resistance exercise [25] and aerobic exercise [74] have been associated with improvements in CVD risk factors. Previous studies of resistance exercise have demonstrated positive effects on blood lipids [25] which may in part be explained by improved insulin sensitivity, and an increase in skeletal muscle lipoprotein lipase activity, resulting in increased VLDL-triacylglycerol catabolism [75].

### Limitations

This is a small study in which only some variables were determined. Habitual dietary intake was not assessed. To be more complete the study should have included determination of insulin levels and sensitivity, as well as albumin bound fatty acids, plasma lipoprotein distribution and fatty acid distribution. In addition, only fasting values of blood variables were determined and the effect of our trial on many CVD risk factors is thus unknown. We also did not account for or make any evaluation of sex or thyroid hormones, catecholamines, glucagon, corticosteroids, all of which known to be metabolic regulators [76-79]. Menstrual cycle phase is known to influence serum lipoproteins [80]. Menstrual cycle was not coordinated with blood collection.

## Conclusions

Although this study was not designed to fully elucidate the mechanisms behind the observed body composition changes, the study has shown that resistance exercise performed twice weekly in overweight women on a low carbohydrate ketogenic may reduce body fat without significantly changing LBM, while resistance exercise on a regular diet may increase LBM in without significantly affecting fat mass. Fasting blood lipids do not seem to be negatively influenced by the combination of a low carbohydrate diet and resistance exercise.

## List of abbreviations

LBM: Lean body mass; DEXA: Dual - Energy X - ray Absorptiometry; TAG: Triacylglycerol; Tot-C: Total cholesterol; HDL-C: High density lipoprotein cholesterol; LDL-C: Low density lipoprotein cholesterol; CVD: Cardiovascular disease.

## Competing interests

The authors declare that they have no competing interests.

## Authors' contributions

PTJ and IAM designed the study and organized and analyzed the data.

ATH and SET provided critical statistical and methodical advice. PTJ, ATH, SET and HDM wrote the manuscript. HDM was in charge of medical safety and interpretation of blood sample values. All authors read and approved the final manuscript.
